# Intake of red and processed meat and risk of renal cell carcinoma: a meta-analysis of observational studies

**DOI:** 10.18632/oncotarget.18549

**Published:** 2017-06-16

**Authors:** Shaojing Zhang, Qingwei Wang, Juanjuan He

**Affiliations:** ^1^ Department of Urology Surgery, The First Affiliated Hospital, Zhengzhou University, Zhengzhou, Henan Province, China; ^2^ Department of Breast Surgery, The First Affiliated Hospital, Zhengzhou University, Zhengzhou, Henan Province, China

**Keywords:** red and processed meat, renal cell carcinoma, meta-analysis, relative risk

## Abstract

**Background:**

Findings on the association between intake of red and processed meat with renal cell carcinoma (RCC) risk are mixed. We conducted a meta-analysis to investigate this association.

**Materials and Methods:**

Eligible studies up to August 31, 2016, were identified and retrieved by searching the MEDLINE and Embase databases along with manual review of the reference lists from the retrieved studies. The quality of the included studies was evaluated using the Newcastle-Ottawa Quality Assessment Scale. The summary relative risk (SRR) and corresponding 95% confidence interval (CI) were calculated using a random-effects model.

**Results:**

Twenty-three publications were included in this meta-analysis: four cohort studies, one pooled study, and 18 case-control studies. The SRR (95% CI) for the highest vs. lowest intake of red meat was 1.36 (1.16–1.58, Pheterogeneity < 0.001); that for processed meat was 1.13 (95% CI, 1.03–1.24, Pheterogeneity = 0.014). Linear dose-response analysis yielded similar results, i.e., the SRR for per 100 g/day increment of red meat and per 50 g/day increment of processed meat was 1.21 (95% CI, 1.08–1.36) and 1.16 (95% CI, 0.99–1.36), respectively. A non-linear association was observed only for red meat (Pnonlinearity = 0.002), and not for processed meat (Pnonlinearity = 0.231). Statistically significant positive associations were observed for intake of beef, salami/ham/bacon/sausage, and hamburger.

**Conclusions:**

This meta-analysis indicates a significant positive association between red and processed meat intake and RCC risk.

## INTRODUCTION

In the United States, the incidence of kidney cancer is the seventh and tenth highest in men and women, respectively [1]. Renal cell carcinoma (RCC) is the most common malignancy of the kidney [2]. Globally, RCC incidence demonstrates regional variations, with age-standardized incidence rates being about 11.9 per 100,000 in developed areas and 2.5 per 100,000 in less developed regions [3]. The incidence of RCC has increased in most countries over the past decade [4]. However, the reasons for the regional and historical variations in RCC incidence are unknown. The demonstrated risk factors for RCC development include age, smoking [5], obesity [6], hypertension [7], and acquired cystic kidney disease [8]. Although data are limited, a family history of kidney cancer [9], certain analgesics [10], history of diabetes [11], and occupational exposure (e.g., asbestos, silica, solder) have been linked to increased risk of RCC [12].

The consumption of red and processed meat has long been recognized as a risk factor of human cancer, as such meats are rich in well-established carcinogens, such as heterocyclic amines (HCAs), polycyclic aromatic hydrocarbons (PAHs), and *N*-nitroso compounds (NOCs) [13, 14]. Many epidemiological studies have investigated the association between the consumption of red and processed meat and the risk of RCC [15–28]. Recently, two meta-analyses [29, 30] of observational studies have been published on this issue. According to the former meta-analysis of 13 case-control studies, Mohammed et al. [30] concluded that there is evidence supporting an independent relation between high consumption of red and processed meat and the incidence of kidney cancer. Whereas findings of the latter one [29], which included 12 case-control, 3 cohort and 1 pooled analysis, were not supportive of an independent relation between red or processedmeat intake and kidney cancer. Since then, numerous epidemiological studies [31–40] evaluating the aforementioned associations have been published and have reported inconsistent results. In addition, the exact form of the dose-risk relationship of these associations has not been clearly defined. To better understand this issue, we carried out a comprehensive meta-analysis of observational studies according to Meta-analysis Of Observational Studies in Epidemiology (MOOSE) guidelines [41].

## MATERIALS AND METHODS

### Data sources and searches

Two investigators (Z.S.J. and H.J.J.) conducted a computerized literature search independently in MEDLINE (from January 1, 1966) and Embase (from January 1, 1974) through to August 31, 2016. We searched the relevant studies using the following words and/or Medical Subject Heading (MeSH) terms: 1) intake OR consumption OR diet OR red meat OR processed meat OR preserved meat OR beef OR pork OR veal OR mutton OR lamb OR ham OR sausage OR bacon; 2) kidney OR renal; 3) carcinoma OR cancer OR neoplasm OR neoplasia; and 4) case-control OR cohort OR prospective OR retrospective. Furthermore, we reviewed the reference lists of the relevant articles to identify additional studies. Only studies published in English were included.

### Study selection

In the present analysis, red meat was defined as beef, veal, pork, lamb, or a combination thereof [22]; processed meat was generally defined as meat products made largely from pork, veal, and beef that undergoes preservation such as curing, smoking, or drying [22]. We also assessed some specific red/processed meats, including beef, pork, hamburger, salami/ham/bacon/sausage, and barbecued/pan-fried/broiled meat. We attempted to evaluate other subcategories that were described as “lamb” and “liver”, but the number of included studies assessing these meats was too limited.

### Studies were included if they

- were published as an original article;
- used a case-control or cohort design;
- reported relative risk (RR) estimates with corresponding 95% CIs for the association between red and/or processed meat intake and the risk of RCC.

Non–peer-reviewed articles, abstracts, commentaries/letters, ecologic assessments, correlation studies, experimental animal studies, and mechanistic studies were excluded. When multiple reports on the same study were available, only the most informative one was considered.

### Data collection and items

A standardized data collection sheet was designed before the extraction. Two investigators (Z.S.J. and H.J.J.) separately extracted the basic information (first author's last name, location, publication year, sample source, duration of follow-up, number of cases and non-cases), data of interest (methods of ascertainment of dietary variables, exposure type [total or individual meats], comparison groups, methods of outcome assessment, RR [95% CI] for the highest vs. lowest level), and adjustments. From each study, we extracted the risk estimates that reflected the greatest degree of control for potential confounders.

### Quality assessment of individual studies

We used the NOS checklist to assess study quality [42], where the quality of case-control and cohort studies is assessed using three parameters: selection (four items, each awarded one star), comparability (one item, which can be awarded up to two stars), and exposure/outcome (three items, each awarded one star). A score of ≥ 7 stars is indicative of a high-quality study.

### Statistical methods

We used a random-effects model to calculate the SRRs (95% CIs) for the high vs. low and dose-response analyses. This model accounts for heterogeneity among studies [43]. As outcomes were relatively rare, the ORs in the case-control studies were considered approximations of RRs. When sex-specific estimates were available, we analyzed for this separately. For studies [16, 18–20, 27, 28, 36, 37, 40] that presented results on meat subtypes separately, but not that for overall red/processed meat, we combined the results using a fixed-effects model, and then included the pooled RR estimates in the meta-analysis.

We used the χ^2^ test to assess heterogeneity among studies, defining significant heterogeneity as *P* < 0.10. We also used the I^2^ statistic to explore the extent of inconsistency, with I^2^ > 50% indicating high heterogeneity and I^2^ < 25% indicating no significant heterogeneity [44]. We performed subgroup and meta-regression analysis on location, study design (case-control vs. cohort), FFQ type (validated vs. non-validated), available exposure data, study quality score, number of cases, and confounders (smoking status, BMI, dietary energy intake, alcohol consumption, intake of vegetables and fruits, history of hypertension). We conducted sensitivity analysis by repeating the meta-analysis of remaining studies after omitting one study at a time.

When possible, we performed linear dose-response meta-analysis per 100 g/day increment of red meat intake and per 50 g/day increment of processed meat intake using generalized least squares trend estimation (GLST) [45, 46]. These methods require that the number of cases and person–time or controls for at least three quantitative exposure categories be known. GLST requires medians for categories of intake levels. For open-ended categories, we assumed that the range was the same as the adjacent interval. When the exposures were expressed as “times” or “servings”, we converted it into grams (g) using 120 g and 50 g as a standard portion size for red meat and processed meat, respectively, as described in the WCRF/AICR report [22]. For the study [34] reporting intakes as g/1000 kcal/day, the intake as g/day was estimated using the average energy intake reported in the article. We performed potential non-linear dose-response analysis using the best-fitting 2-term fractional polynomial regression model [47]. A likelihood ratio test was used to assess the difference between the non-linear and linear models to test for non-linearity [47]. All statistical analyses were performed using R-package (Version 2.11.0 beta, R Development Core Team, NJ, USA) and Stata version 11.0 (StataCorp, College Station, TX, USA). A 2-sided test with α = 0.05 was used to indicate the level of significance.

## RESULTS

### Search results and study characteristics

**Figure 1:**
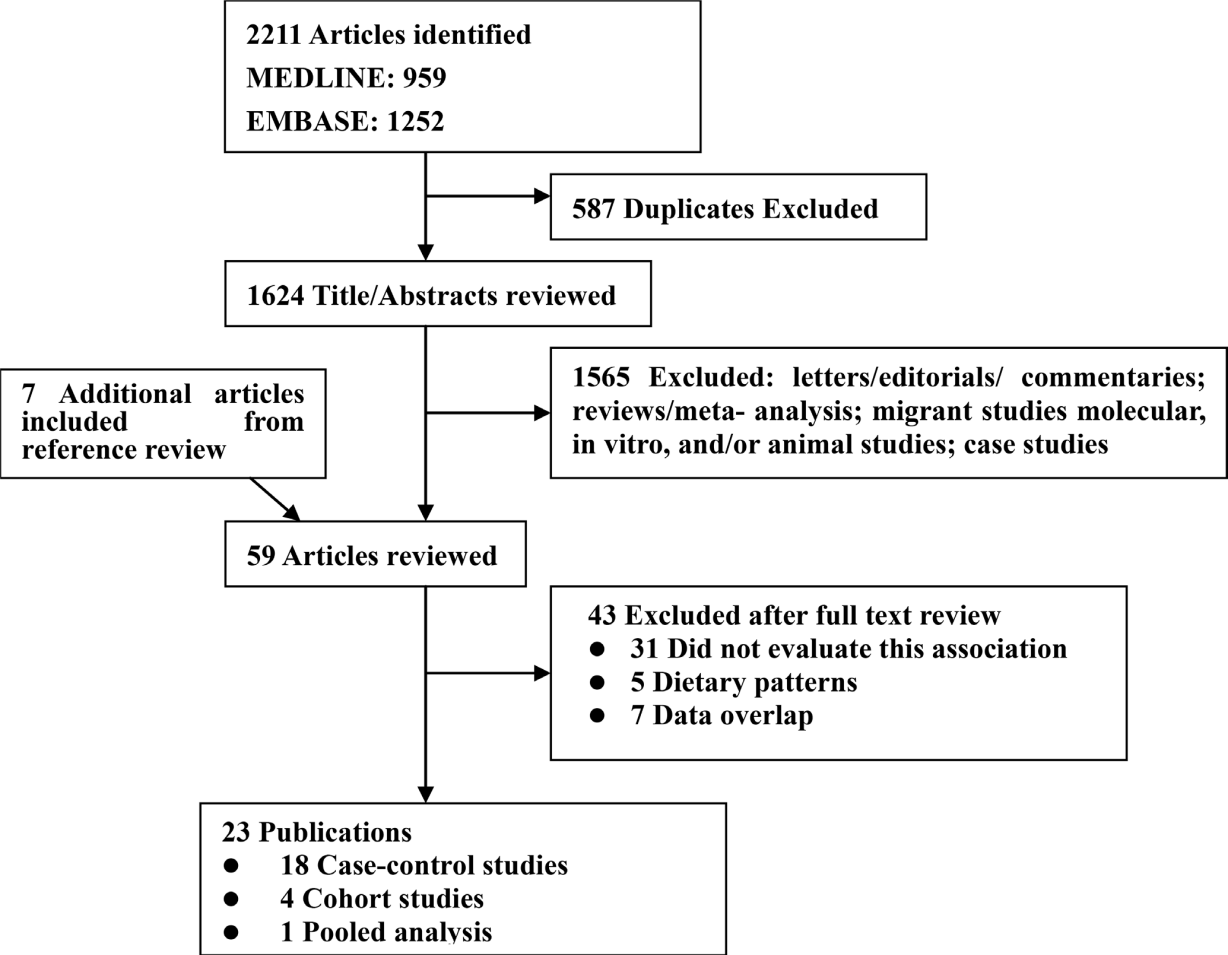
Flow diagram of systematic literature search on red and processed meat intake and renal cell carcinoma risk

The search strategy generated 2,211 citations, of which 59 were considered of potential value and for which the full text was retrieved for detailed evaluation. An additional seven articles were identified from a review of the references. Forty-three of these 66 articles were subsequently excluded from the meta-analysis. The studies by Di Maso et al. [48] and Bravi et al. [17] were based on the same data. We included the latter [17] because it had the most informative data. The studies by De Stefani et al. [23] and De Stefani et al. [33] were based on the same setting, but in different time periods, i.e., from 1988 to 1995 and from 1996 to 2004. Therefore, we included both studies. We also included two studies with overlapping reports [19, 35]: one on overall processed meat intake [35] and the other on red meat intake [19]. One pooled study included 13 independent cohorts [15]; another four cohort studies included four different cohorts (the European Prospective Investigation into Cancer and Nutrition study [EPIC] [32]; the NIH-AARP Diet and Health Study [34], the Japan Collaborative Cohort Study for Evaluation of Cancer Risk [JACC] Study [18], and California Seventh-day Adventists [28]). An eventual total 23 publications were included in this meta-analysis (Figure 1).

The characteristics of these 23 publications are described in Tables 1 and 2. They comprised four prospective cohort studies [18, 28, 32, 34], one pooled study [15], and 18 case-control studies [16, 17, 20–27, 31, 33, 35, 37–40]. A total 14,285 patients with RCC and 1,821,615 controls/participants were included. The studies were conducted in North America (*n* = 11), Europe (*n* = 7), Asia (*n* = 1), and South America (*n* = 3). The pooled study was conducted in the United States and in Europe. The methods used in all studies for assessing meat consumption were based on the food items semiquantitative Food Frequency Questionnaire (FFQ). The Newcastle-Ottawa Scale (NOS) scores ranged 5–9; 19 studies were deemed to be of high quality (≥ 7 stars) (Supplementary Table 1).

**Table 1:** Characteristics of case-control studies of red and processed meat intake and renal cell carcinoma risk

Author/ year/ Country	number of subjects enrolled	Outcome determined	Dietary assessments	Exposure (Highest vs. lowest)	RR (95% CI) (Highest vs. lowest)	Adjustments	Score
Hospital-based							
Melkonian et al. 2016, USA [31]	659 RCC cases 699 controls	Histological	Self-administered Validated FFQ	Red meat T3 vs. T1	2.28 (1.67–3.10)	Age, sex, BMI, history of hypertension, smoking status, total energy intake, total fruit and vegetable intake	7
De Stefani et al. 2012 Uruguay [33]	144 RCC 2,532 Controls	Histological	Validated FFQ-64 Interview	Processed meat: >28.3 vs. 11.4 g/d	1.21 (0.65–2.25)M 2.15 (0.90–5.13)W	Age, residence, BMI, smoking status, smoking, alcohol drinking, mate consumption, total energy, total vegetables and fruits, total white meat	7
Aune et al. 2009, Uruguay [40]	114 RCC 2,032 Controls	Histological	Validated FFQ-64 Interview	Red meat: 300.2 vs. 85.5 g/d Beef: 300 vs. 85.5 g/d Lamb: 150 vs. 0g/d Processed meat:	2.72 (1.22–6.07) 2.53 (1.14–5.59) 0.77 (0.22–2.67) 1.23 (0.68–2.22)	Age, sex, residence, education, income, interviewer, smoking status, alcohol, dairy foods, grains, fatty foods, fruits and vegetables, fish, poultry, mate drinking, BMI and energy intake	7
Bravi et al. 2007, Italy [17]	767 RCC 1,534 Controls	Histological	Interview FFQ-78 validated	Red meat: 5.9 vs. 2.4 serving/wk Processed meat:3.9 vs.0.9 serving/wk	0.84 (0.62–1.14) 0.64 (0.45–0.90)	Age, center, sex, period of interview, education, smoking, alcohol drinking, BMI, family history of kidney cancer, total energy intake	7
Hsu et al. 2007, Europe [16]	1,065 RCC 1,509 Controls	Histological	Interview FFQ-23 validated	Red meat: ≥ 1 time/wk vs. < 1 time/month Ham, salami, sausages ≥ 1 time/wk vs. < 1 time/month	2.01 (1.02–3.99) 1.03 (0.71–1.51)	Age, country, sex, smoking, education, BMI, hypertension medication use, alcohol consumption, total white meat consumption	7
Tavani et al. 2000, Italy [21]	190 RCC 7,990 Controls	Histological	Self-administered FFQ, NA	Red meat: > 6 vs. ≤ 3 servings/wk	1.1 (0.8–1.6)	Age, year of recruitment, sex, education, smoking, alcohol, fat, fruit and vegetable intakes.	5
De Stefani et al. 1998, Uruguay [23]	121 RCC 243 Controls	Histological	Interview FFQ-23 validated	Red meat: > 365 vs. ≤ 208 g/d Barbecued: > 53 vs. ≤ 12 g/d Processed meat: > 53 vs. ≤ 12 g/d	3.42 (1.76–6.65) 2.07 1.03–4.19 0.78 (0.45–1.39)	Age, sex, residence, urban-rural status, education, BMI, mate drinking.	6
Talamini et al. 1990, Italy [26]	240 RCC 665 Controls	Histological	Interview FFQ NA	Salami: ≥ 3 serving/wk vs. the lowest	1.01 (0.63–1.61) 1.25 (0.85–1.85)	Age, sex, education, area of residence, BMI	5
**Population-based**							
Hu et al. 2011, Canada [35]	1,345 RCC 5,039 Controls	Histological	Validated FFQ-69 Interview	Processed meat: ≥ 5.42 vs.0.94 servings/wk	1.3 (1.1–1.6)	Age, province, education, BMI, sex, alcohol use, smoking, total vegetable and fruit intake, and total energy intake	9
Daniel et al. 2011, USA [36]	1,192 RCC 1,175 Controls	Histological	Interviewer Diet History Questionnaire	Red meat: 42.0 vs.11.7 g/1000kal/d Barbecued meat:16.7 vs.0 g/1000kal/d Pan-fried meat: 15.6 vs.0.3 g/1000kal/d Broiled meat:7.6 vs.0 g/1000kal/d	1.11 (0.83–1.48) 1.35 (1.01–1.79) 1.05 (0.80–1.38) 0.75 (0.59–0.96)	Age, race, sex, education, smoking status, BMI, history of hypertension, family history of cancer, alcohol, intake of fruit and vegetables, total energy intake, and other meat intake and/or cooking method offsets	8
Brock et al. 2009, USA [39]	323 RCC 1,820 Controls	Histological	Self-administered questionnaire NA	Red meat: > 1.7 vs.0–0.8 servings/d Cured meat: > 0.6 vs.0–0.1servings/d	1.5 (1·0–2.4) 1.6 (1·1–2.5)	Age, sex, smoking, obesity, hypertension, physical activity, alcohol and vegetable intake and tea and coffee consumption	9
Grieb et al. 2009, USA [37]	335 RCC 337 Controls	Histological	Interview FFQ-70 Validated	Red meat: > 5 vs. < 1 time/wk Bacon and sausage: > 5 vs. < 1 time/wk	4.43 (2.02–9.75) 1.28 (0.63–2.62)	Age, sex, race, income, BMI, smoking	8
Hu et al. 2003, Canada [19]	1,279 RCC 5,380 Controls	Histological	Self-administered FFQ-70 Validated	Beef, pork or lam:T3 vs.T1 Hamburger:T3 vs.T1 Bacon:T3 vs.T1 Sausage:T3 vs.T1	1.3 (1.0–1.6) 1.4 (1.1–1.8) 1.3 (1.0–1.6) 1.5 (1.2–2.0)	Age, sex, province, education, BMI, alcohol use, smoking and total energy	
Handa et al. 2002, Canada [20]	461 RCC 672 Controls	Histological	Self-administered FFQ-69 NA	Beef: Q4 vs. Q1	1.2 (0.7–2.0) M	Age, smoking status, BMI	7
Yuan et al. 1998, USA [22]	1204 RCC 1204 Controls	Histological	Interview FFQ-40 NA	Processed meat: Q4 vs. Q1	1.15 (0.86–1.54)	level of education, BMI, history of hypertension cigarettes, analgesics, use of amphetamines	7
Wolk et al. 1996, multi centers [24]	1,185 RCC 1,526 Controls	Histological	Self-administered and interview FFQ, NA	Red meat: Q4 vs. Q1 Processed meat: Q4 vs. Q1	0.94(0.73–1.20) 0.94(0.73–1.22)	Age, sex, stud center, BMI, smoking, total calories	7
Chow et al. 1994, USA [25]	690 RCC 707 Controls	Histological	Self-administered FFQ-65 validated	Red meat: > 9.3 vs.4.3 servings/wk Processed meat:5.0 vs. 1.4 servings/wk	1.3 (0.9–1.9) 1.0 (0.7–1.5)	Age, sex, cigarette smoking, and BMI.	8
Maclure et al. 1990, USA [27]	203 RCC 604 Controls	Histological	Interview FFQ Validated	Beef: Q4 vs. Q1 Pork: Q4 vs. Q1 Bacon: Q4 vs. Q1 Processed meat: Q4 vs. Q1	3.4(1.6–7.2) 0.74(0.4–1.4) 0.85(0.47–1.5) 1.3(0.86–2.0)	Age, sex	7

Abbreviation: BMI, body mass index; FFQ, food frequency questionnaire; NA, not available; M, men, W, women; RCC, renal cell carcinoma.

**Table 2:** Characteristics of cohort studies of red and processed meat intake and renal cell carcinoma

Author/year, Country	Study name and number of subjects FU, yr	Case ascertainment Cases (*n*)	Dietary assessments	Exposure details	RR (95% CI) (Highest vs. lowest)	Adjustments	Score
Rohrmann et al. 2015 [32], Europe	EPIC *N* = 375,851 FU, 11.6 yr	cancer or mortality registries 691 RCC	Self-administered Validated FFQ	Red meat: > 80 vs. 0–9.9 g/d Processed meat: > 80 vs. 0–9.9 g/d	1.46 (0.99–2.15) 1.23 (0.84–1.79)	Age, center, sex, education, BMI, history of hypertension, smoking status, duration of smoking, energy intake,alcohol consumption, fruit and vegetable consumption	9
Daniel et al. USA2012 [34]	NIH-AARP Diet and Health Study *N* = 491,841 FU, 9 yr	cancer registry 1,816 RCC	Self-administered Validated FFQ-124	Red meat:48.1 vs. 6.8 g/1000k/d Processed meat:19.9 vs. 1.4 g/1000k/d	1.08 (0.92–1.28) 1.12 (0.95–1.32)	Age, sex, education, race, marital status, family history of any cancer, BMI, smoking status, hypertension, diabetes, alcohol, total energy, legumes, whole grains	9
Lee et al. 2008, Europe and USA [15]	13 cohorts *N* = 774,952 FU, 7-20 yr	medical records, cancer registries 1,478 RCC	Self-administered Validated FFQ	Red meat: > 80 vs. < 20 g/d Processed meat:12–27 vs. < 4 g/d	0.99 (0.85–1.16) 1.06 (0.88–1.28)	Age, history of hypertension, BMI, smoking, combination of parity and age at first birth, fruit and vegetable consumption, alcohol intake, and total energy intake	9
Washio et al. 2005, Japan [18]	JACC *N* = 114,517 FU, 10yr	mortality registries 48 RCC	Self-administered Validated questionnaire	Beef: 1–2 vs. seldom times/wk Pork: 1–2 vs. seldom times/wk Ham and sausage: 1–2 vs. seldom times/wk	1.73(0.74–4.08) 0.92(0.34–2.27) 1.16(0.42–3.24)	Age, sex	7
Fraser et al. 1990, USA [28]	California Seventh-day Adventists *N* = 34,198 FU, 6.2 yr	mortality registries 14 RCC	Self-administered Validated FFQ	Beef: > 1 vs. < 1 serving/wk	1.59 (0.49–5.01)	Age, sex	6

Abbreviation: EPIC, the European Prospective Investigation into Cancer and Nutrition; JACC, the Japan Collaborative Cohort Study for Evaluation of Cancer Risk Study; BMI, body mass index; FFQ, food frequency questionnaire; NA, not available, RCC, renal cell carcinoma; FU, follow-up.

### Red meat

### High vs. low analysis

**Figure 2:**
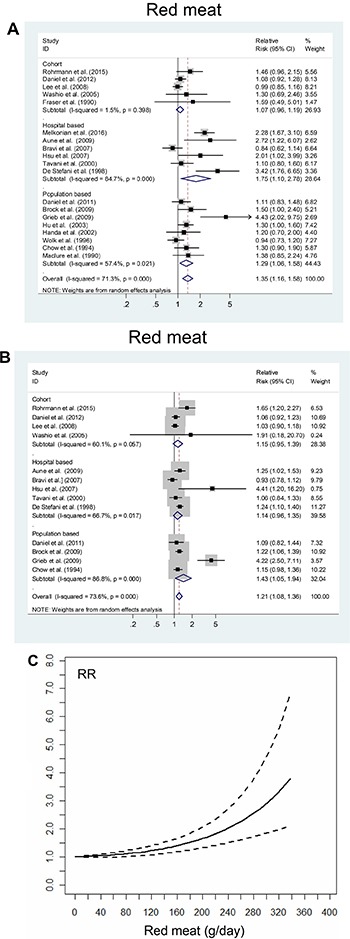
The summary risk association between red meat intake and risk of renal cell carcinoma according to (**A**) the highest vs. lowest analysis; (**B**) linear dose-response analysis (Per 100 g/day increment); (**C**) non-linear dose-response analysis. Studies are sub-grouped according to design.

Nineteen studies reported on the highest vs. lowest levels of red meat intake and RCC risk. The summary relative risk (SRR) was 1.36 (95% confidence interval [CI], 1.16–1.58); there was evidence of high inter-study heterogeneity (P_heterogeneity_ < 0.001, I^2^ = 71.3%; Figure 2A).

### Dose-response analysis

Thirteen studies were included in the dose-response analysis of red meat intake and RCC risk. The SRR per 100 g/day increment was 1.21 (95% CI, 1.08–1.36), with evidence of high heterogeneity (P _heterogeneity_ < 0.001, I^2^ = 73.6%; Figure 2B). There was evidence of a non-linear association of red meat intake and RCC risk (*P* = 0.002). Visual inspection of the curve suggested that the risk increased linearly up to approximately 240 g/day red meat intake. Above that, the risk increase became even steeper (Figure 2C).

### Processed meat

### High vs. low analysis

**Figure 3:**
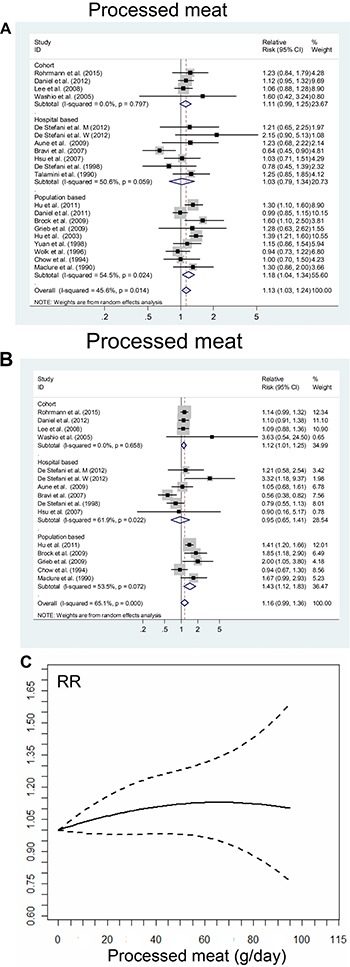
The summary risk association between processed meat intake and risk of renal cell carcinoma according to (**A**) the highest vs. lowest analysis; (**B**) linear dose-response analysis (Per 50 g/day increment); (**C**) non-linear dose-response analysis. Studies are sub-grouped according to design.

Nineteen studies reported on the highest vs. lowest level of processed meat intake and RCC risk. The SRR was 1.13 (95% CI, 1.03–1.24), and there was evidence of moderate inter-study heterogeneity (P_heterogeneity_ = 0.014, I^2^ = 45.6%; Figure 3A).

### Dose-response analysis

Fourteen studies were included in the dose-response analysis of processed meat intake, and the SRR per 50 g/day increase was 1.16 (95% CI, 0.99–1.36), and there was high inter-study heterogeneity (P_heterogeneity_ < 0.001, I^2^ = 65.1%; Figure 3B). There was no evidence of a non-linear association between processed meat intake and RCC risk (*P* = 0.231; Figure 3C).

### Subgroup, meta-regression, and sensitivity analyses

Table 3 shows the results of the stratified and meta-regression analyses. For high vs. low consumption of red meat, we observed an increased risk of RCC in case-control studies (SRR = 1.46; 95% CI, 1.18−1.81), but not in cohort studies (SRR = 1.07; 95% CI, 0.96−1.19). The SRRs were significant for studies conducted in North America (SRR = 1.44; 95% CI, 1.17–1.76; I^2^ = 68.6%) and South America (SRR = 3.12; 95% CI, 1.87–5.20; I^2^ = 72.3%), but not in those conducted in Europe (SRR = 1.04; 95% CI, 0.86–1.26) and Asia (SRR = 1.30; 95% CI, 0.69–2.46). There was significant between-subgroup heterogeneity in stratified analysis of location (*P* for difference = 0.038). Adjustments by body mass index (BMI), smoking, history of hypertension, total energy intake, intake of vegetables and fruits, and alcohol consumption did not significantly change the SRR for RCC risk.

**Table 3:** Subgroup analyses of red and processed meat intake and renal cell carcinoma risk, high *vs*. low

Sub-groups	Red meat					Processed meat				
	Studies, n	SRR (95% CI)	*P* for heterogeneity	I^2^ (%)	*P* for difference	Studies, *n*	SRR (95% CI)	*P* for heterogeneity	I^2^ (%)	*P* for difference
All	19	1.36 (1.16–1.58)	< 0.001	71.3		19	1.13 (1.03–1.24)	0.014	45.6	
Design					0.751					0.956
Cohort	5	1.07 (0.96–1.19)	0.398	1.5		4	1.11 (0.99–1.25)	0.797	0	
Case-control	14	1.46 (1.18–1.81)	< 0.001	75.0		14	1.13 (1.00–1.27)	0.004	55.4	
Sources of control					0.470					0.152
Population-based	8	1.29 (1.06–1.58)	0.021	57.4		9	1.18 (1.04–1.34)	0.024	54.5	
Hospital-based	6	1.75 (1.10–2.78)	< 0.001	84.7		6	1.03 (0.79–1.34)	0.059	50.6	
Geographic locations					0.038					0.178
Europe	6	1.04 (0.86–1.26)	0.069	51.2		5	0.98 (0.78–1.23)	0.065	54.7	
USA	10	1.44 (1.17–1.76)	0.001	68.6		9	1.20 (1.07–1.34)	0.062	46.2	
South America	2	3.12 (1.87–5.20)	< 0.001	72.3		3	1.16 (0.81–1.67)	0.275	22.7	
Asia	1	1.30 (0.69–2.460	-			1	1.60 (0.58–4.44)	-		
Data available					0.189					0.424
Self-administered	12	1.25 (1.07–1.45)	0.002	63.5		8	1.17 (1.03–1.33)	0.069	46.7	
Interview	7	1.79 (1.16–2.75)	< 0.001	78.8		11	1.09 (0.95–1.26)	0.052	43.7	
Type of FFQ					0.294					0.857
Validated	16	1.44 (1.20–1.75)	< 0.001	75.8		15	1.12 (1.00–1.25)	0.012	49.9	
Not available	4	1.10 (0.90–1.33)	0.317	15.0		4	1.17 (0.94–1.44)	0.172	40.0	
Study quality score										
High (NOS score > 6)	16	1.32 (1.30–1.54)	< 0.001	71.5	0.464	17	1.13 (1.03–1.25)	0.011	48.4	0.713
Low (NOS score ≤ 6)	3	1.78 (0.79–4.00)	0.012	77.4		2	1.03 (0.66–1.63)	0.177	45.1	
Adjustments										
BMI, yes	15	1.39 (1.16–1.66)	< 0.001	77.4	0.705	17	1.12 (1.01–1.24)	0.008	50.2	0.426
no	4	1.22 (0.95–1.57)	0.842	0		2	1.34 (0.91–1.98)	0.713	0	
Smoking, yes	15	1.30 (1.11–1.52)	< 0.001	73.1	0.302	15	1.12 (1.01–1.25)	0.006	53.4	0.979
no	4	1.75 (1.10–2.79)	0.130	47.0		4	1.17 (0.92–1.50)	0.441	0	
Energy intake, yes	8	1.21 (1.01–1.46)	< 0.001	77.9	0.167	10	1.11 (1.01–1.26)	0.001	65.2	0.716
no	11	1.49 (1.15–1.93)	0.001	68.0		9	1.17 (1.01–1.35)	0.637	0	
Hypertension, yes	7	1.35 (1.07–1.70)	< 0.001	78.7	0.929	7	1.09 (1.00–1.18)	0.457	0	0.947
no	12	1.38 (1.10–1.73)	< 0.001	68.2		12	1.13 (0.97–1.32)	0.009	54.6	
Consumption of vegetables and fruits, yes	7	1.40(1.07–1.85)	< 0.001	79.2	0.883	7	1.17 (1.03–1.34)	0.152	34.6	0.368
No	12	1.33 (1.09–1.63)	< 0.001	67.4		12	1.08 (0.94–1.24)	0.012	54.5	
Alcohol, yes	10	1.17 (1.02–1.34)	0.030	51.2	0.151	11	1.15 (1.01–1.30)	0.002	62.4	0.635
No	9	1.65 (1.18–2.31)	< 0.001	77.2		8	1.08 (0.94–1.24)	0.684	0	

For high vs. low consumption of processed meat, we observed a borderline significant risk of RCC in both case-control (SRR = 1.13; 95% CI, 1.00−1.27; I^2^ = 55.4%) and cohort studies (SRR = 1.11; 95% CI, 0.99−1.25; I^2^ = 0). The SRR was significant for studies conducted in North America (SRR = 1.20; 95% CI, 1.07–1.34), but not for studies conducted in South America, Europe, and Asia.

In univariate meta-regression analysis, only location was a significant factor for the association between red meat intake and RCC risk; however, no variables were significant factors for processed meat intake.

The estimation of overall homogeneity and the effect of removing one study at a time from the analysis confirmed the stability of the relationship between intake of red and processed meat and RCC risk (data not shown). In addition, repeat analysis of high vs. low intake using the studies included in the linear dose-response analysis yielded results similar to that of the original analysis (red meat: SRR = 1.20; 95% CI, 1.07–1.34; processed meat: SRR = 1.13; 95% CI, 1.00–1.27).

### Publication bias

**Figure 4:**
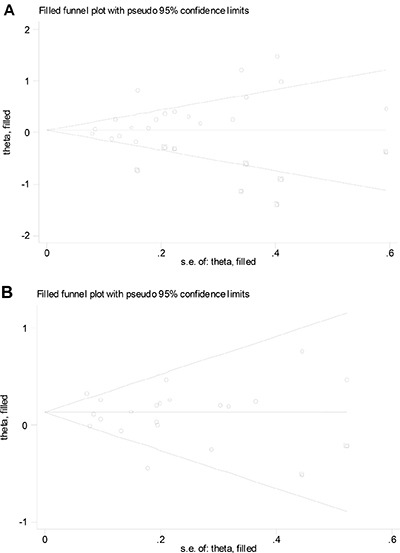
Filled funnel plot of log relative risk vs. standard error of log relative risks in studies that evaluated the effect of red meat (**A**) and processed meat (**B**) intake on the risk of renal cell carcinoma.

For intake of red meat, visual inspection of the funnel plot, as well as Egger's test (*P* = 0.087) and Begg's test (*P* = 0.005), indicated publication bias. The trim-and-fill method indicated that eight additional risk estimates were needed to balance the funnel plot (Figure 4A), and the summary risk estimates were again not significant (SRR = 1.09; 95% CI, 0.92–1.29). For intake of processed meat, visual inspection of the funnel plot, as well as Egger's test (*P* = 0.145) and Begg's test (*P* = 0.183), did not indicate publication bias. The trim-and-fill method indicated that two additional risk estimates were needed to balance the funnel plot (Figure 4B), and the summary risk estimates were unchanged (SRR = 1.12; 95% CI, 1.02–1.23).

### Individual meat items

There were positive associations between RCC risk with the consumption of beef (SRR = 1.89; 95% CI, 1.25–2.86), hamburger (SRR = 1.41; 95% CI, 1.12–1.78), and ham/salami/bacon/sausage (SRR = 1.30; 95% CI, 1.16–1.47). RCC risk was not positively associated with the intake of pork or barbecued/pan-fried/broiled meat (Supplementary Table 2).

## DISCUSSION

The results of this comprehensive meta-analysis show that the consumption of red and processed meat is associated with increased RCC risk, as per the high vs. low and linear dose-response meta-analyses. There was significant heterogeneity across studies for both red and processed meat intake. In non-linear models, RCC risk appeared to increase approximately linearly with increased intake of processed meat, whereas there was evidence of non-linear increased risk with increased intake of red meat. Among individual red and processed meat types, there were statistically significant positive associations for the intake of beef, salami/ham/bacon/sausage, and hamburger.

Several mechanisms have been proposed to explain how the consumption of red and processed meat enhances cancer risk, and include the high intake of proteins and fats and intake of carcinogens (e.g., NOCs, HCAs, PAHs) [49, 50]. A large prospective cohort study observed increased risk of RCC with high consumption of nitrate and nitrite, the precursor of NOCs, and total RCC (hazard ratio = 1.28, 95% CI, 1.10–1.49) [51]. In animal studies, benzo (a) pyrene (BaP) and PhIP were two of the most potent PAHs [52]. Epidemiological studies have found a positive association between BaP and PhIP and RCC [34, 36]. The high saturated fat content of red and processed meat has also been proposed as a culprit for the increased risk of RCC in some studies [53], but not in other studies [54, 55].

In comparison with previous meta-analyses [29, 30], the present updated analysis included an additional 11 studies (two updated studies), and a total 14,285 patients with RCC and 1,821,615 controls/participants, which can provide sufficient power for detecting the putative moderate associations. In addition, we conducted comprehensive analyses based on high vs. low, linear, and non-linear dose-response models; importantly, we performed rigorous quality assessment. We also explored the association between specific subtypes of meat and RCC risk. Finally, by conducting a meta-regression analysis, we could explore the source of heterogeneity between studies.

We found that red and processed meat consumption was significantly associated with increased risk of RCC in the case-control studies, which might drive the overall epidemiological findings of the present study, but not in the cohort studies. Case-control studies are more susceptible to recall and selection bias than are cohort studies, as lifestyles and diet habits in retrospective case-control studies are determined after the diagnosis of cancer. Although the meta-regression results suggested that study design did not significantly alter the aforementioned associations, we observed that the positive association was weaker in the cohort studies than in the case-control studies. Therefore, the finding that red and processed meat consumption is associated with increased RCC risk should be received with caution.

The present meta-analysis has several limitations. First, inaccurate assessments of dietary intake could have led to overestimations of the range of intakes and consequent underestimation of the magnitude of the aforementioned relationship [56, 57]. Not all studies used validated semiquantitative FFQs for dietary assessment; however, subgroup analyses showed that the use of validated vs. non-validated FFQs did not significantly affect the association between the consumption of red and processed meat and RCC risk. Although some FFQs were not validated, its reproducibility has been confirmed, with the correlation coefficients between the two assessments being 0.77 and 0.55 for red meat and processed meat, respectively [58]. In addition, analyses of the highest vs. lowest intake are limited because they do not account for true differences among studies. For example, the definition of lowest intake of red meat ranged from 0 to < 1 time/month [16], and the highest intake ranged from 1 time/week [16] to > 365 g/day [23].

Second, there was great inter-study heterogeneity. Stratified and meta-regression analyses revealed a significant positive association between studies from North America (but not from Europe), and study location was the only significant factor in the association between intake of red meat and RCC risk. This might be attributed to the fact that different populations consume different types, levels of meat, and their cooking practices differ, which may partly explain the high heterogeneity among the included studies. Additionally, there was considerable heterogeneity in the dose-response analysis models, which might be ascribed to a consequence of the conversions of the intake units.

Third, the residual confounders inherent in primary observational studies are always of concern. Although most of the included studies reported adjusted risk estimates of RCC for confounders, some appeared to have failed to fully control for confounders. For example, only seven studies used adjustments for history of hypertension, which is one of the established risk factors of RCC [7]. High intake of red meat and processed meat is likely to be associated with other unhealthy lifestyle choices, for example, smoking, obesity, and lower intake of vegetables and fruits, all of which are indicated as risk factors for RCC [5, 6]. In addition, alcohol consumption is common in people with high intake of red and processed meat, and moderate alcohol consumption was identified as a protective factor against RCC [59]. When we limited the meta-analysis to studies controlled for BMI, smoking, alcohol use, and intake of vegetables and fruits, the aforementioned positive associations were not significantly modified.

Fourth, HCA and PAH formation increases with cooking temperature and duration; however, data on the degree of meat doneness in the included studies were not available. Additionally, the non-linear trend with intake of red meat should be interpreted with caution due to the low statistical power in the extremes of red meat intake distribution. This is an issue of the fractional polynomial method. Most of the included studies were based on data from Western populations; additional research in other populations is warranted to generalize these findings.

Lastly, we acknowledge the presence of significant publication bias in the results for red meat intake. The overall risk estimates for the association for red meat consumption were probably an overestimation, as small studies with null results tend not to be published. Indeed, the trim-and-fill method indicated that eight additional risk estimates were needed to balance the funnel plot, and the summary risk estimates were attenuated and not statistically significant.

In conclusion, our limited data suggest that high intake of red and processed meat may increase RCC risk. However, because the effect was only found in case-control studies and might be a consequence of bias, confounding factors, and importantly, publication bias, further prospective epidemiological studies that control for possible confounders and that examine the association between meat consumption and RCC risk are required.

## SUPPLEMENTARY MATERIALS AND TABLES


