# Correction: Intake of red and processed meat and risk of renal cell carcinoma: a meta-analysis of observational studies

**DOI:** 10.18632/oncotarget.25715

**Published:** 2018-06-22

**Authors:** Shaojin Zhang, Qingwei Wang, Juanjuan He

**Affiliations:** ^1^ Department of Urology Surgery, The First Affiliated Hospital, Zhengzhou University, Zhengzhou, Henan Province, China; ^2^ Department of Breast Surgery, The First Affiliated Hospital, Zhengzhou University, Zhengzhou, Henan Province, China

**This article has been corrected:** The correct spelling of author name is given below:

**Shaojin Zhang**

Original article: Oncotarget. 2017; 8:77942-77956. https://doi.org/10.18632/oncotarget.18549

