# Supplementary material for: Intake of red and processed meat and risk of renal cell carcinoma: a meta-analysis of observational studies
Source: Oncotarget. 2017 Jun 16;8(44):77942–56. doi: 10.18632/oncotarget.18549 (PMC5652826; doi:10.18632/oncotarget.18549)
Supplement: Supplementary file 1 [file oncotarget-08-77942-s001.pdf]

Intake of red and processed meat and risk of renal cell carcinoma: a meta-analysis of observational studies

SUPPLEMENTARY MATERIALS

Supplementary Table 1: Quality assessment according to the Newcastle-Ottawa scale

Author	Year	Selection	Comparability	Outcome	Exposure	Score
**Case-control**						
Melkonian et al.	2016	3	2	/	2	7
De Stefani et al.	2012	2	2	/	3	7
Hu et al.	2011	4	2	/	3	9
Daniel et al.	2011	4	2	/	2	8
Brock et al.	2009	4	2	/	3	9
Grieb et al.	2009	4	2	/	2	8
Aune et al.	2009	2	2	/	3	7
Bravi et al.	2007	2	2	/	3	7
Hsu et al.	2007	2	2	/	3	7
Hu et al.	2003	4	2	/	3	9
Handa & Kreiger	2002	3	2	/	2	7
Tavani et al.	2000	2	1	/	2	5
De Stefani et al.	1998	2	1	/	3	6
Yuan et al.	1998	4	2	/	1	7
Wolk et al.	1996	3	2	/	2	7
Chow et al.	1994	3	2	/	3	8
Maclure et al.	1990	4	1	/	2	7
Talamini et al.	1990	3	1	/	1	5
**Cohort**						
Rohrmann et al.	2015	4	2	3	/	9
Daniel et al.	2012	4	2	3	/	9
Lee et al.	2009	4	2	3	/	9
Washio et al.	2005	3	1	3	/	7
Fraser et al.	1990	2	1	3	/	6

Supplementary Table 2: Meat subtype and risk of renal cell carcinoma

Subtype of meat	No. of studies	SRR (95% CI)	*P*	I^2 (%)
Beef	5	1.89 (1.25–2.86)	0.215	31.0
Pork	2	0.79 (0.47–1.33)	0.708	0
Barbecue/grilled meat /broiled meat	2	1.12 (0.79–1.59)	0.003	78.2
Bacon/sausages/ham/salami	6	1.30 (1.16–1.47)	0.729	0
Hamburger	2	1.41 (1.12–1.78)	0.848	0
